# Prevalence of human respiratory syncytial virus, parainfluenza and adenoviruses in East Africa Community partner states of Kenya, Tanzania, and Uganda: A systematic review and meta-analysis (2007–2020)

**DOI:** 10.1371/journal.pone.0249992

**Published:** 2021-04-27

**Authors:** Therese Umuhoza, Wallace D. Bulimo, Julius Oyugi, Jean Pierre Musabyimana, Alison A. Kinengyere, James D. Mancuso

**Affiliations:** 1 Institute of Tropical and Infectious Diseases, University of Nairobi, Nairobi, Kenya; 2 Department of Emerging Infectious Diseases, United States Army Medical Directorate – Africa, Nairobi, Kenya; 3 School of Medicine, University of Nairobi, Nairobi, Kenya; 4 Medical Research Center, Rwanda Biomedical Center, Kigali, Rwanda; 5 Sir Albert Cook Library, College of Health Sciences, University Makerere, Kampala, Uganda; 6 Department of Preventive Medicine and Biostatistics, Uniformed Services University of the Health Sciences, Bethesda, Maryland, United States of America; International Islamic University, PAKISTAN

## Abstract

**Background:**

Viruses are responsible for a large proportion of acute respiratory tract infections (ARTIs). Human influenza, parainfluenza, respiratory-syncytial-virus, and adenoviruses are among the leading cause of ARTIs. Epidemiological evidence of those respiratory viruses is limited in the East Africa Community (EAC) region. This review sought to identify the prevalence of respiratory syncytial virus, parainfluenza, and adenoviruses among cases of ARTI in the EAC from 2007 to 2020.

**Methods:**

A literature search was conducted in Medline, Global Index Medicus, and the grey literature from public health institutions and programs in the EAC. Two independent reviewers performed data extraction. We used a random effects model to pool the prevalence estimate across studies. We assessed heterogeneity with the I^2^ statistic, and Cochran’s Q test, and further we did subgroup analysis. This review was registered with PROSPERO under registration number CRD42018110186.

**Results:**

A total of 12 studies met the eligibility criteria for the studies documented from 2007 to 2020. The overall pooled prevalence of adenoviruses was 13% (95% confidence interval [CI]: 6–21, N = 28829), respiratory syncytial virus 11% (95% CI: 7–15, N = 22627), and parainfluenza was 9% (95% CI: 7–11, N = 28363). Pooled prevalence of reported ARTIs, all ages, and locality varied in the included studies. Studies among participants with severe acute respiratory disease had a higher pooled prevalence of all the three viruses. Considerable heterogeneity was noted overall and in subgroup analysis.

**Conclusion:**

Our findings indicate that human adenoviruses, respiratory syncytial virus and parainfluenza virus are prevalent in Kenya, Tanzania, and Uganda. These three respiratory viruses contribute substantially to ARTIs in the EAC, particularly among those with severe disease and those aged five and above.

## Introduction

Acute respiratory tract infections (ARTIs) are among the five most common causes of morbidity and mortality globally, accounting for approximately 3.9 million deaths annually. Most of these deaths occur among young children in developing countries [1]. Viruses are responsible for a large proportion of ARTIs and these are associated with various syndromes of the upper and lower respiratory tract, including: acute otitis media, croup, pneumonia, bronchiolitis, and asthma [2, 3]. Additionally, co-infections of viruses and bacteria are commonly reported in severe cases of ARTIs [4, 5]. Although viral aetiologies are associated with a large percentage of acute respiratory tract infections, it is difficult to link specific viral agents to a specific syndrome. This is due to the complexity and broad spectrum of illnesses caused by these pathogens. Furthermore, the emergence of new viral strains and cost of diagnosis contribute to the inability to detect viral agents in order to associate these pathogens with a specific syndrome. However, influenza viruses, one of the major causative agents of acute respiratory tract infections, have been extensively studied with an established global surveillance program. This program was set up to assess the threat of the emergence of strains which could cause pandemic disease [6].

Globally, non-influenza respiratory viruses have received less attention respiratory virus surveillance programs, hence few studies are available in the published literature [7]. Nevertheless, a few previous studies have indicated the risk of non-influenza viruses to public health, and that some viral families have the potential to cause epidemics [8]. The non-influenza respiratory viruses most commonly associated with ARTIs include human respiratory syncytial viruses (HRSV), parainfluenza viruses (HPIVs), and adenoviruses (HAdV), among others [9]. The consequences of these respiratory viruses result in an enormous direct and indirect economic burden on public health. In the United States alone, the estimated annual economic burden of non-influenza viral respiratory tract infections is equivalent to $40 billion [10]. Interestingly, a global incidence of at least 33.1 million has been associated with HRSV in young children under five [11]. The same study indicated a mortality range of 48,000–74,500 for children younger than 5 years and estimated that 99% of these deaths occurred in developing countries [11].

In Sub-Saharan Africa, recent annual incidence data of community-acquired pneumonia is estimated to be 131 million, with significant proportion of these aetiologies due to viruses [12]. A study conducted in Senegal reported that a range of respiratory viruses cause influenza-like illness (ILI) with substantial proportions due to influenza viruses (53.1%; 1045/1967), rhinoviruses (30%; 591/1967), enteroviruses (18.5%; 364/1967), and HRSV (13.5%; 266/1967) in children under five years old [13]. A review of the aetiology of ARTIs in children <5 years in Sub-Saharan Africa showed that HRSVs, HPIVs, and HAdV were among the leading causes of ARTIs [14]. Moreover, in 2018 a systematic review and meta-analysis of HRSV prevalence in Africa reported an overall HRSV prevalence of 14%, thus indicating that this pathogen contributes significantly to severe respiratory illness on the continent [15].

The World Health Organization Regional Office for Africa (WHO-AFRO) 2012 country profiles indicated that acute lower respiratory infections (ALRTIs) were amongst the top three causes of death in the East African Community (EAC). In EAC partner states, the proportionate mortality from lower respiratory tract infections (LRTIs) was: Tanzania (8.7%), Kenya (12.3%), Uganda (9.6%), South Sudan (12%), Rwanda (10%) and Burundi (12.5%). Amongst these EAC states, Kenya, Tanzania, Uganda, and Rwanda have established surveillance programs for influenza and other respiratory viruses which are recognized by the WHO [16]. The EAC has a large population with approximately 161 million inhabitants [17] who are highly mobile and integrated, with a common regional market, tourism, and social and cultural exchange. These factors increase the risk of infectious disease transmission and spread in the EAC, as has been described elsewhere [18]. Several other studies have demonstrated various viral aetiologies as causes of ARTIs in the EAC [19, 20]. In Kenya, a study conducted at the Kilifi district hospital reported a high prevalence (34%) of HRSV infections in young children which was associated with severe pneumonia [21]. Human parainfluenza, adenoviruses, and other respiratory viruses were also reported [21]. The use of molecular techniques have also enhanced the detection and identification of other non-influenza respiratory viruses in countries with no consistent respiratory disease surveillance programs [22].

A large number of programs for surveillance of influenza-like illness (ILI) and severe acute respiratory illness (SARI) were established in order to strengthen health security after the establishment of the 2005 International Health Regulations. However, such programs have primarily estimated the occurrence of influenza viruses and have either not assessed or not reported other (non-influenza) viruses [23]. This leaves an inconsistent assessment of the epidemiology of non-influenza viruses in the EAC region. This review addresses some of this gap by providing a systematic review of the published and unpublished literature of pooled prevalence of HRSV, HPIV, and HAdV among symptomatic patients in EAC partner states over the period between 2007 and 2020. These three viruses were the most frequent non-influenza respiratory viruses detected in the surveillance programs; the prevalence of other non-influenza viruses was rarely reported, precluding formal systematic review.

## Methods

### Eligibility criteria

This review considered studies that reported laboratory-confirmed infections caused by HRSV, HPIV, and HAdV in all age groups. These three respiratory viruses are among those frequently reported to cause respiratory tract infections other than influenza. In addition, the review included a broad range of study participants, including those with acute respiratory tract infections (ARTIs), influenza-like illnesses (ILIs), severe acute respiratory illnesses (SARIs), and other syndromes including pneumonia. Studies reporting asymptomatic infections were excluded in this review.

The review considered observational studies, including prospective and retrospective cross-sectional and cohort studies that were either descriptive, analytical, or both. Case series, individual case reports, letters to editors, reviews, commentaries, and qualitative studies were excluded. Only studies published in English, including those from unpublished reports from the grey literature, were included. Published studies and unpublished reports documented in the period between 1^st^ January 2007 and 31^st^ December 2020 were included.

### Search strategy

An initial unlimited search was conducted in Medline that allowed more refined search strategies tailored for Global Index Medicus. The initial search was performed by verification of the text words contained in the title and abstract of the index terms, which were used to describe the articles using keywords and Medical Subject Heading (MeSH) terminologies (S1 File). This informed the development of a search strategy that was used for each information source. In addition, reference lists of all studies selected for inclusion were screened for additional relevant publications. The search was first completed in 2019 then updated using the same methodology in 2021.

To obtain information from the grey literature, inquiries regarding these viruses were made directly to the ministries of health. We also searched the databases of government medical research institutions, teaching hospitals, and university libraries in EAC partner states. Electronic database search or author correspondence was performed with the Kenya Medical Research Institute (KEMRI) and Kenyatta National Hospital (KNH) for Kenya; National Institute for Medical Research (NIMR) for Tanzania; Uganda Virus Research Institute (UVRI) and Makerere University (MAK), and Mulago Hospital (MUH) for Uganda; Institut National de Sante Public (INSP) for Burundi; and Rwanda Biomedical Center (RBC) for Rwanda. In addition, other non-government public health research programs in the East African Community were contacted.

All identified citations were collated and uploaded into Zotero software, version 5.0, (Corporation for Digital Scholarship, Vienna, VA) and duplicates were removed. Titles and abstracts were re-screened against the eligibility criteria, and studies that met the eligibility criteria were retrieved in full and their details imported into JBI SUMARI (Joanna Briggs Institute, Adelaide, Australia). The full texts of selected studies were assessed in detail against the eligibility criteria by two parallel reviewers, and any disagreements were resolved by a third investigator.

### Data extraction and management

Prior to data extraction, all selected studies were critically appraised for methodological quality. This was accomplished with a standardized critical appraisal instrument from the Joanna Briggs Institute by two independent reviewers. This review followed the guideline of systematic reviews of prevalence and incidence manual with the use of JBI SUMARI software available at //www.jbisumari.org.

All data extracted from the selected studies were included in the review using a standardized data extraction tool in JBI SUMARI software. Extracted data consisted of: name of the primary author, year of publication, locality, age categories of participants, clinical characteristics, study design, length of the study period, specimen type, laboratory test type, number of cases, and total population. Age categories were reported as under five years only, five and above only, or all ages. Clinical conditions or syndromes were recorded as influenza-like illness (ILI) only, severe acute respiratory illness (SARI) only, or acute respiratory tract infections (ARTIs) for studies which investigated both ILI and SARI. Pneumonia was categorized as a severe acute respiratory illness (SARI). Study durations were classified as five months or less, six to twelve months, and more than twelve months.

### Risk of bias assessment

The risk of bias was assessed in the selected studies through the use of an eight variable rating scale [24–27]. Each was given a score for how well-defined and clearly reported the variable was. The variables included: i) length of a study period which was at least three months to permit laboratory processing of samples and analysis, ii) year of documentation or publication, iii) study area (country or study locality), iv) age group description, v) clinical condition or syndrome using a standard case definition, vi) standardized type of specimen collection, vii) laboratory methodology and viii) type of study design. Each variable received a score of one for a clear and defined record and zero for missing or unclear documentation. Thus, the scores could range from 0 to 8. We categorized scores of 0 to 2 as high risk for bias, 3 to 5 as medium risk, and 6 to 8 as low risk.

### Data synthesis and analysis

Data were analysed using Stata^®^13 (StataCorp, College Station, TX). The dataset was re-organized and coded for analysis, and further meta-analysis was performed using the “metaprop” package in STATA program [28]. Initially, unadjusted prevalence of HRSV, HPIV and HAdV infections were calculated based on the crude numerators and denominators found among the individual studies.

To ensure that studies with very small or large prevalence were kept in the overall estimates, the Freeman-Tukey double-arcsine transformation technique was performed using the metaprop command [29]. This procedure stabilized the variance of study-specific prevalence before applying a random-effects model (RE) to assess heterogeneity and generate a pooled prevalence estimate. The random-effects model allowed the effect to vary across studies, providing more conservative estimates with wider confidence intervals given the observed heterogeneity between studies [30]. It was implemented using the method of DerSimonian and Laird [31], whereas 95% confidence intervals (CIs) were drawn from exact binomial distribution (Clopper-Pearson) [32]. The I^2^ statistic, Cochran’s Q test, and subgroup analysis were used to assess heterogeneity [33]. The statistical values of I^2^ expressed the variation of in-between studies differences as a percentage, simplifying the interpretation with the “rule of thumb” [33, 34]. Generally, a substantial heterogeneity was indicated by the values of I^2^ >50%, whereas a tentative categories of minimal (I^2^< = 25%), low (I^2^ = 25–4950%), moderate (I^2^ = 50–75%), and high (I^2^> = 75%)[33, 35]. A funnel plot was generated and an egger test was performed with a metabias command to evaluate for publication bias [36]. The Egger test of P<0.10 indicated a significant publication bias [37–39].

Prevalence of infection was described by country, age group, and clinical conditions. In addition, pie charts were used to display the prevalence of HRSV, HPIV and HAdV infections in the region with quantum geographical information system (qGIS). Subgroup analyses were performed on variables of public health importance including: clinical condition (ILI, SARI, or ARTIs), age groups (below five, five and above, or all ages), and locality (Kenya, Tanzania, or Uganda). We followed guidelines for systematic reviews of prevalence and incidence from the Joanna Briggs Institute to accomplish this review. In addition, the Preferred Reporting Items for Systematic Reviews and Meta-Analyses (PRISMA) guided the report writing (S2 File). This review was registered in the International Prospective Register of Systematic Reviews (PROSPERO) under registration number CRD42018110186.

## Results

### Review records

**Fig 1:**
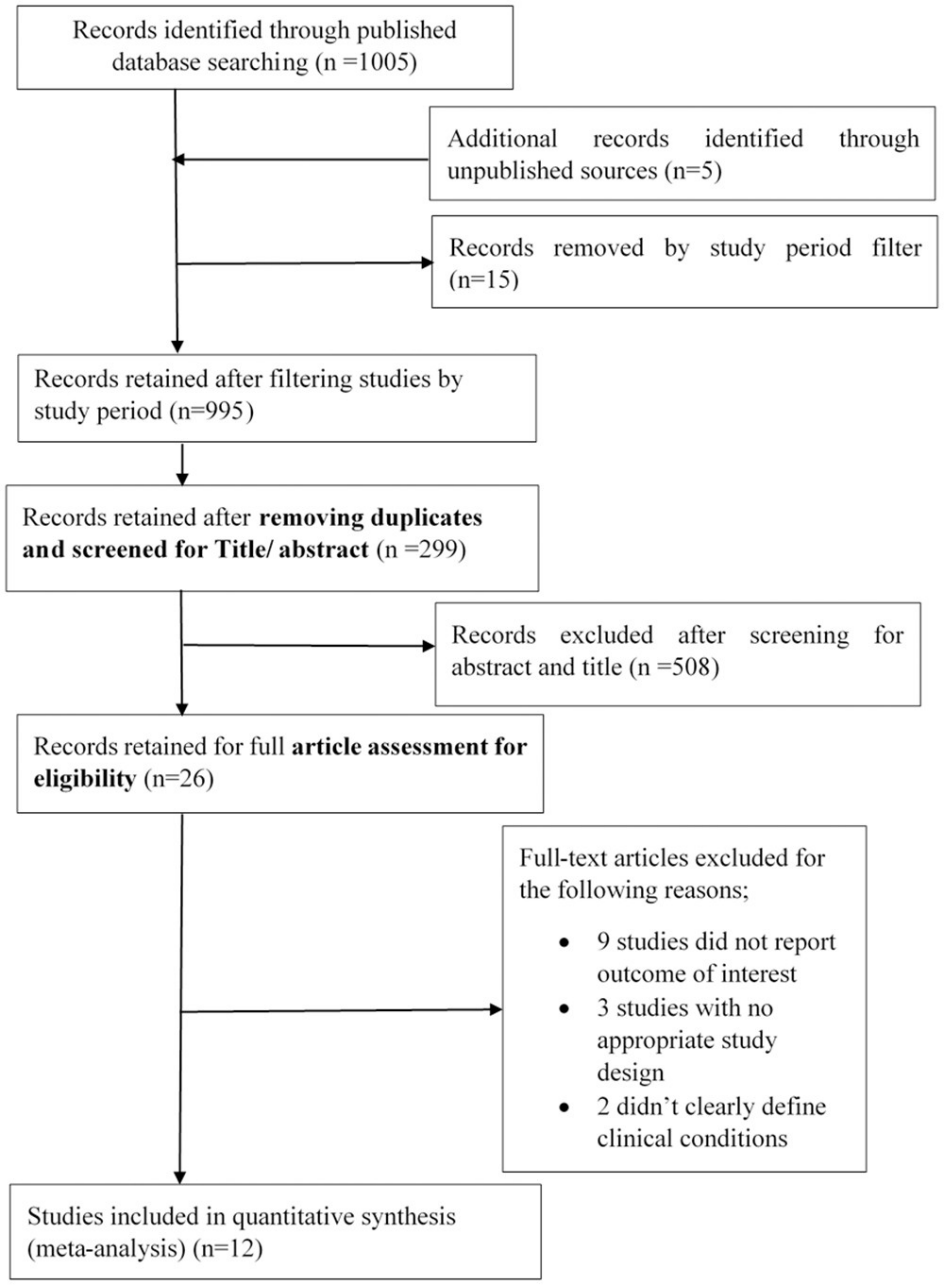
Review records.

In this review, we found 1005 records in published databases and 5 reports from unpublished sources. After filtering based on the defined study period (2007–2020), 995 (990 published and 5 unpublished) studies were retained. A total of 299 (294 published and 5 unpublished) were retained after removing 188 duplicates. After screening using the abstract and title, we excluded 508 which did not meet eligibility criteria. The remaining 26 studies were assessed for full article eligibility, and 14 were excluded for different reasons shown (Fig 1). Twelve (12) studies met eligibility criteria and were therefore included in the study with further meta-analysis.

### Characteristics of selected studies

In this review, qualified studies were documented from 2009 to 2018 (S3 File). A large number of studies on the selected viruses were conducted in Kenya (Table 1). These comprised studies of HRSV (n = 8, 80%), HPIV (n = 6, 75%) and HAdV (n = 7, 77.7%) (S4 File). Tanzania and Uganda had one study each which reported results for all three of the viruses under investigation. There were no available studies from Rwanda, Burundi, and South Sudan that assessed any of the three viruses during the period under review. Most studies reported for the three countries were cross-sectional. Furthermore, the majority of studies (n = 6) were of acute respiratory tract infections (ARTIs) involving both ILI and SARI. A few studies (n = 4) reported ILI only, and 2 were SARI only. Five studies reported having enrolled individuals of all ages, while four studies recruited participants aged under five years only, and one study exclusively enrolled participants aged five years and older. The majority (n = 7) of studies were conducted over a period of >12 months, two were <5 months, and one was 6–12 months. Polymerase chain reaction (PCR) was the most common diagnostic test, and most studies collected and analysed both oropharyngeal swabs (OPS) and nasopharyngeal swabs (NPS) specimens. In general, the studies had a low risk of bias.

**Table 1:** Study characteristics.

Outcomes	HRSV		HPIV		HAdV	
Study	Number (n)	Proportion (%)	Number (n)	Proportion (%)	Number (n)	Proportion (%)
**Locality**						
Kenya	8	80	6	75	7	77.7
Tanzania	1	10	1	12.5	1	11.1
Uganda	1	10	1	12.5	1	11.1
**Clinical Condition**						
ILI	2	20	2	25	2	22.2
SARI	2	20	1	62.5	2	22.2
ARTIs*	6	60	5	12.5	5	55.5
**Population**						
Under Five	3	30	1	12.5	2	22.2
Five and above	1	10	1	12.5	1	11.1
All ages**	5	50	4	50	5	55.5
**Study design**						
Cross-sectional	7	70	6	75	7	77.7
Cohort	2	20	1	12.5	1	11.1
**Study Period**						
Short-term (≤ months)	2	20	2	25	2	22.2
Medium-term (6–12 months)	1	10	1	12.5	1	11.1
Long-term (>12 months)	5	50	3	37.5	4	44.4
**Lab Test**						
Virus isolation	1	10	2	25	2	22.2
PCR	9	90	6	75	7	77.7
**Specimens**						
OPS	1	10	1	12.5	1	11.1
NPS	2	20	3	37.5	3	33.3
OPS and NPS	7	70	4	50	5	55.5
**Risk of Bias**						
Low risk	9	90	7	87.5	8	88.8
Moderate risk	1	10	1	12.5	1	11.1

*ARTIs studies of both ILI and SARI.

**All ages included uncategorized age groups.

OPS = oropharyngeal swabs, NPS = nasopharyngeal swabs, PCR = polymerase chain reaction, ARTI = acute respiratory tract infection, ILI = influenza-like illness, SARI = severe acute respiratory illness, HRSV = human respiratory syncytial virus, HPIV = human parainfluenza virus, and HAdV = human adenovirus.

### Prevalence of human respiratory syncytial virus, parainfluenza and adenoviruses

**Fig 2:**
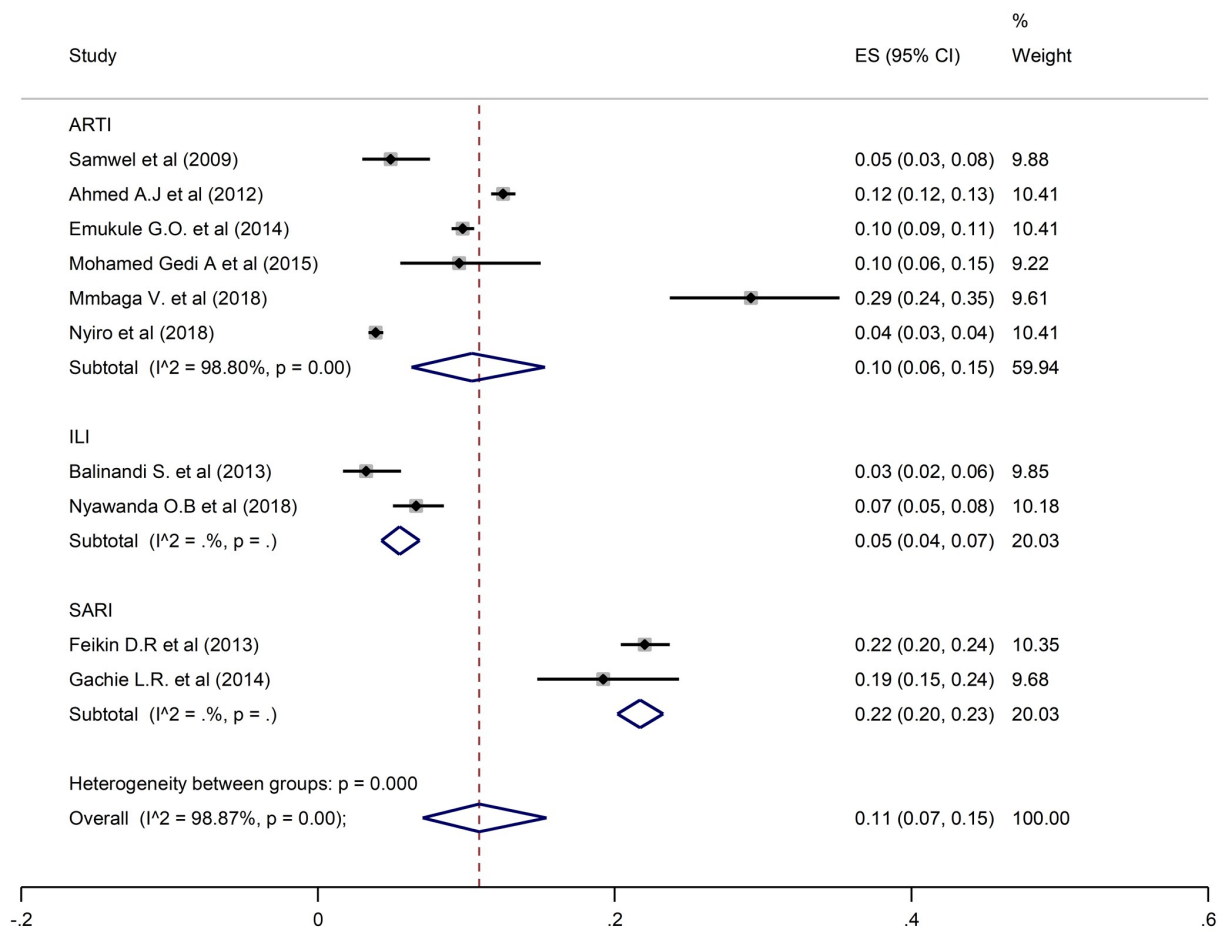
Pooled prevalence of HRSV in ARTIs, ILI, and SARI.

The overall pooled prevalence of HRSV was 11% (95% CI: 7–15) reported from 10 studies with a total population of 22,627 participants (Fig 2).

**Fig 3:**
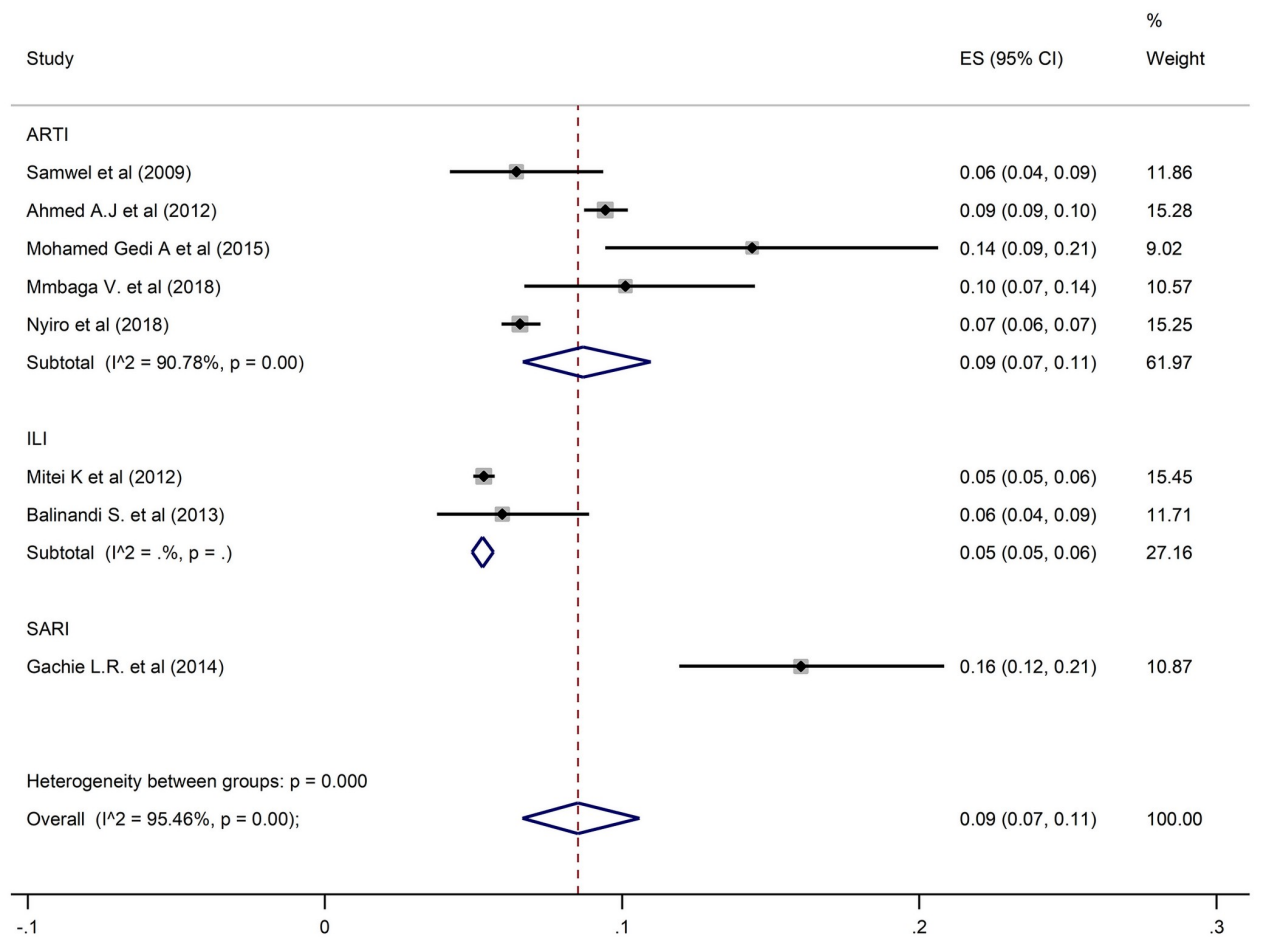
Pooled prevalence of HPIV in ARTIs, ILI, and SARI.

The estimated overall pooled prevalence of 9% (95% CI: 7–11) HPIV was estimated from 8 studies with 28,363 participants (Fig 3).

**Fig 4:**
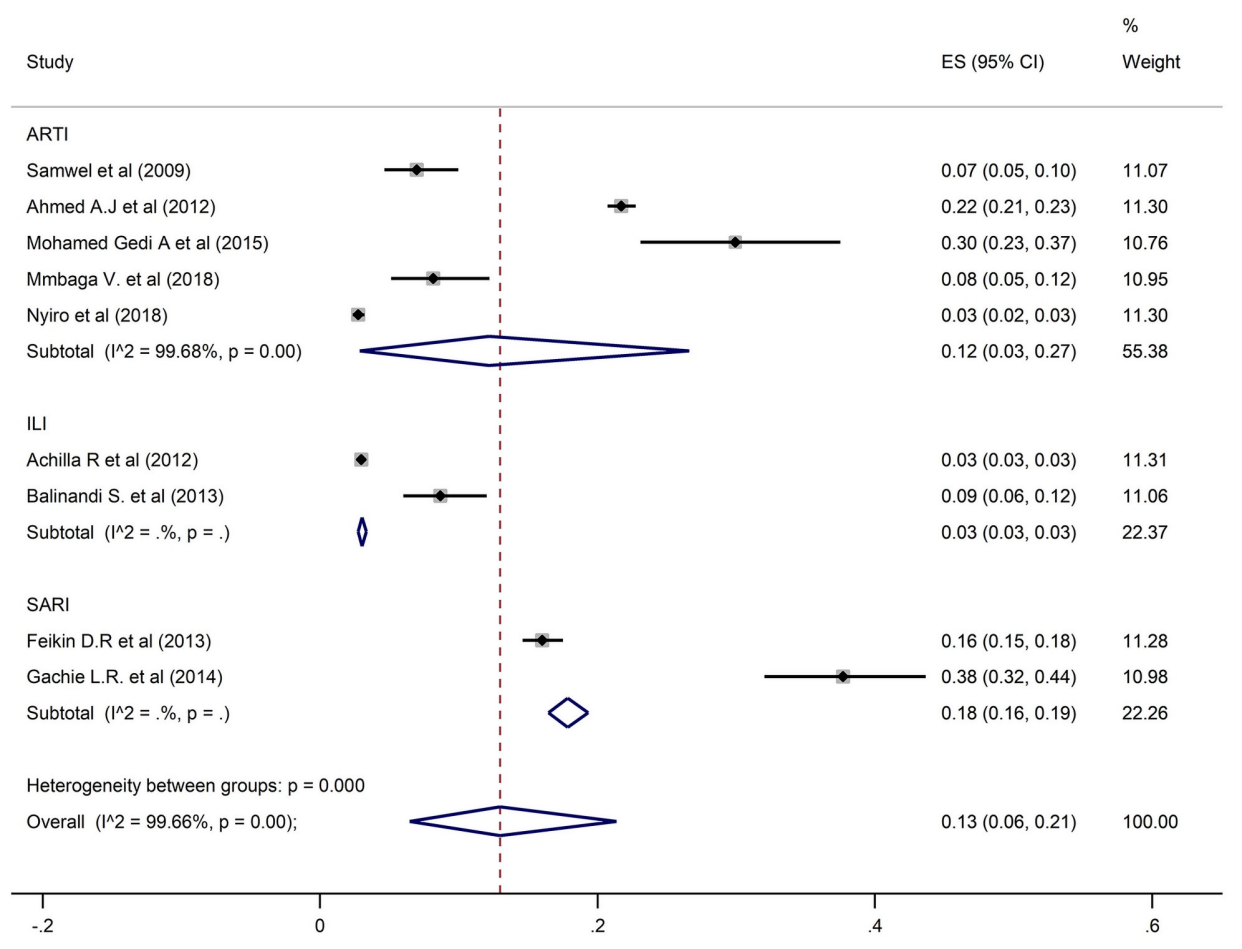
Pooled prevalence of human adenoviruses in ARTIs, ILI, and SARI.

HAdV overall pooled prevalence was 13% (95% CI: 6–21) recorded in 9 studies with 28,829 participants (Fig 4).

**Fig 5:**
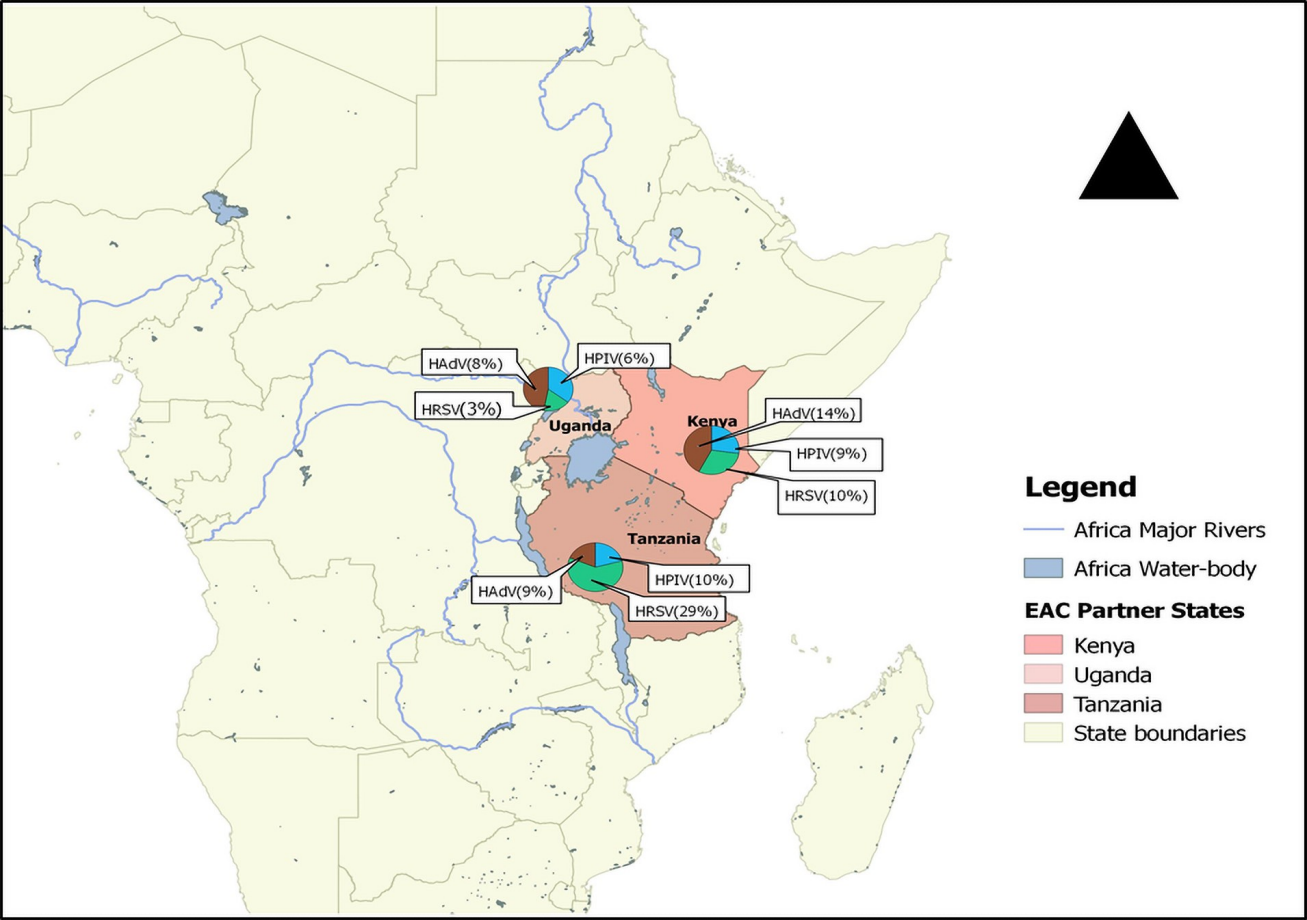
Distribution of HRSV, HPIV and HAdV in three EAC states between 2007 and 2020.

Substantial heterogeneity in the pooled prevalence of the three viruses was seen in the included studies, according to severity of illness, age group, and locality. Prevalence of the three viruses differed in the three countries (Fig 5).

A pooled prevalence of 10% (6–15; 95% CI) was found for HRSV, 9% (7–11; 95% CI) for HPIV, and 12% (3–27; 95% CI) for HAdV when restricting the analysis to the studies that investigated ARTIs. Prevalence estimates from ILI studies only found HRSV, HPIV and HAdV prevalences of 5% (95% CI: 4–7, 5% (95% CI: 5–6), and 3% (95% CI: 3–3.5) respectively. In contrast, estimates from SARI studies only were higher for all three viral pathogens at 22% (95% CI: 20–23) for HRSV, 16% (95% CI: 12–21) for HPIV and 18% (95% CI: 16–19) for HAdV.

Studies which considered participants of all ages had an estimated prevalence of 9% (95% CI: 5–14), 9% (95% CI: 6–12) and 12% (95% CI: 4–24) for HRSV, HPIV, and HAdV, respectively. Prevalences of 10% (95% CI: 2–24) for HRSV, 6% (95% CI: 4–9) for HPIV, and 15% (95% CI: 13–16) for HAdV were reported in studies that enrolled participants who were under five years of age. In the studies that involved individuals five years and above, HSRV prevalence was similar at 10% (95% CI: 6–15), whereas the prevalence of HPIV and HAdV was much higher at 14% (95% CI: 9–21) and 30% (95% CI: 23–37) respectively.

For studies carried out in Kenya, prevalences of HRSV, HPIV and HAdV were 10% (95% CI: 6–15), 9% (95% CI: 7–11) and 14% (95% CI: 7–25) respectively. In Tanzania, estimated prevalence of HAdV and HPIV were similar at 9% (95% CI: 6–12) and 10% (95% CI: 7–14), whereas HRSV prevalence was higher at 29% (95% CI: 24–35). In the studies conducted in Uganda, the corresponding prevalences of HRSV, HPIV, and HAdV were lower at 3% (95% CI: 2–6), 6% (95% CI: 4–9), and 8% (95% CI: 5–12).

There was no publication bias suggested by the funnel plot and or the Egger’s test (Table 2). Whereas considerable heterogeneity was noted overall and in subgroup analysis, no publication bias was recorded in analysis of subgroups with enough studies to assess (ARTIs, All ages, and Kenya). A similar prevalence to the overall prevalence was documented when restricting the analysis to studies of ARTI: 12% (95% CI: 3–27) for HAdV, 10% (95% CI: 6–15) for HRSV, and 9% (95% CI: 7–11) for HPIV. In addition, studies that involved participants of all ages had a pooled prevalence of 12% (95% CI: 4–24) for HAdV, 9% (95% CI: 5–14) for HRSV, and 9% (95% CI: 6–12) for HPIV. Studies performed in Kenya reported prevalence of 14% (95% CI: 7–25), 10% (95% CI: 6–15), and 9% (95% CI: 7–11) for HAdV, HRSV, and HPIV respectively.

**Table 2:** Summary statistics of selected respiratory viruses’ prevalence.

Outcome	HRSV							
Groups	Studies (n)	Cases (n)	Pop (N)	Crude Prev. (95%CI)	Pooled Prev. (95%CI)	I^2^ (%)	P-value (Egger)	P-value (hetero-geneity)
**Overall**	10	2358	22627	10 (10–11)	11 (7–15)	98.87	0.618	**<0.0001**
**Syndromes**								
ARTIs	6	1685	18619	9 (8–9)	10 (6–15)	98.8	0.654	**<0.0001**
ILI	2	69	1230	5 (4–7)	5 (4–7)			
SARI	2	604	2778	22 (20–23)	22 (20–23)			
**Age**								
All age	5	1641	18456	9 (8–9)	9 (5–14)	98.94	0.914	**<0.0001**
Under Five	3	626	3746	16 (15–18)	10 (2–24)			
Five and above	1	16	168	9 (5–15)	10 (6–15)	-	-	**-**
**Locality**								
Kenya	8	2271	22001	10 (10–11)	10 (6–15)	99	0.763	**<0.0001**
Tanzania	1	75	257	29 (23–35)	29 (24–35)	-	-	**-**
Uganda	1	12	369	3 (1–5)	3 (2–6)	-	-	**-**
	**HPIV**							
**Overall**	8	1905	28363	7 (6–7)	9 (7–11)	95.4	0.179	**<0.0001**
**Syndromes**								
ARTIs	5	1037	12723	8 (7–8)	9 (7–11)	90.7	0.681	**<0.0001**
ILI	2	823	15359	5	5 (5–6)			
SARI	1	45	281	16 (12–20)	16 (12–21)	-	-	**-**
**Age**								
All age	4	1029	12561	8 (8–9)	9 (6–12)	94.3	0.706	**<0.0001**
Under five	1	25	388	6 (4–9)	6 (4–9)	-	-	**-**
Five and above	1	24	167	14 (9–21)	14 (9–21)	-	-	**-**
**Locality**								
Kenya	6	1857	27737	6 (6–7)	9 (7–11)	96.6	0.205	**<0.0001**
Tanzania	1	26	257	10 (7–14)	10 (7–14)	-	-	**-**
Uganda	1	22	369	6 (4–9	6 (4–9)	-	-	**-**
	**HAdV**							
**Overall**	9	2537	28829	9 (8–9)	13 (6–21)	99.66	0.337	**<0.0001**
**Syndromes**								
ARTIs	5	1614	12723	13 (12–13)	12 (3–27)	99.68	0.994	**<0.0001**
ILI	2	417	13328	3	3 (3–3.5)			
SARI	2	506	2778	18 (17–20)	18 (16–19)			
**Age**								
All age	5	2039	25520	8 (7–8)	12 (4–24)	99.8	0.516	**<0.0001**
Under Five	2	427	2885	15 (13–16)	15 (13–16)			
Five and above	1	50	167	30 (23–37)	30 (23–37)	-	-	**-**
**Locality**								
Kenya	7	2484	28203	9 (8–9)	14 (7–25)	99.74	0.307	**<0.0001**
Tanzania	1	21	257	8 (5–12)	8 (5–12)	-	-	**-**
Uganda	1	32	369	9 (6–12)	9 (6–12)	-	-	**-**

N: population, prev.: prevalence, CI: confidence interval, I^2^ (index value): the variation in effect sizes attributable to heterogeneity, P: probability value, ARTI = acute respiratory tract infection, ILI = influenza-like illness, SARI = severe acute respiratory illness, HRSV = human respiratory syncytial virus, HPIV = human parainfluenza virus, and HAdV = human adenovirus.

## Discussion

Most of the EAC partner states have, in collaboration with WHO, established programs for surveillance for ILI and/or SARI in order to strengthen global health security under the 2005 IHR. Kenya was the first country to initiate an influenza surveillance program in 2006, followed by Uganda (2007), Rwanda (2008), and Tanzania (2009) [40, 41]. There is no known surveillance program in South Sudan or Burundi [16]. In this systematic review and meta-analysis, only data from Kenya, Tanzania, and Uganda were available.

The overall pooled prevalence of HRSV was 11%, 9% for HPIV and 13% for HAdV, but there was substantial heterogeneity by severity of illness, age group, and location. Overall, most (80%) of the reported studies were done in Kenya. Most studies were assessed as low risk for bias, and no publication bias was evident.

In this meta-analysis, the overall prevalence of HAdV was 13%. This prevalence is slightly higher than the 9.8% reported in individual studies in the Eastern Mediterranean region [42]. A study conducted in 2017 by Niang *et al*. [13] in Senegal reported 30.8% of HAdV prevalence in people with ILI. This is much higher than the 3% recorded in this review among cases of ILI. In contrast, we obtained a pooled prevalence of 30% of HAdV in individuals of five years and above. This figure is higher than the 15% reported by Holly *et al*. [43] in 2018 among college students in the United States. Moreover, our study found that those under five years had a 15% HAdV prevalence, whereas a study conducted in India among children with SARI found a HAdV prevalence of 8.8% [44]. The differences in prevalence seen between studies is likely attributable in large part to the heterogeneity in the study populations according to severity, age, and location. The variation in prevalence may also be due to of the small number of studies included in this systematic review, the methodology of sampling, or the different diagnostic tests used in the various studies.

In this systematic review, the pooled prevalence of HRSV was 11%, which is similar to a previous estimate of 14.6% among people with ARTIs in Africa [15]. In a 2015 review, the pooled HRSV prevalence among patients with ARTI in China was estimated at 18.7% [45], which was higher than that reported in our meta-analysis. In the same review, the prevalence of HRSV was 22% among SARI patients, which was the same as our results in that population [45]. However, while the study from China demonstrated a higher prevalence among infants (26.5%), our data found the same HRSV prevalence among those under five and those five-years and above (10%).

The overall prevalence of HPIV in this review was 9%. Previous studies have reported generally similar prevalence estimates of HPIV. In Cameroon, HPIV prevalence of 7.5% was reported in 2012 among ILI patients [46], which was slightly higher than the 5% prevalence found among ILI patients in this review. A slightly lower HPIV prevalence estimate of 3.2% has been reported in Latin America [47]. Our analysis found an HPIV prevalence of 16% among patients with SARI, which was higher than that reported among similar patients in China (4.8%) [48]. The higher prevalence of HPIV we found among patients aged five years and above supports previous findings of higher HPIV prevalence among older age groups [49–52].

This systematic review is subject to several limitations. The systematic searches performed in this review were limited to the most accessible and widely used databases of medical literature, including Medline and Global Index Medicus. In addition, the search was complemented with unpublished literature from major public health institutions and research programs in the EAC. We identified several significant sources of heterogeneity in the estimates of pooled prevalence of HRSV, HPIV, and HAdV, including disease severity, age group, and location. Heterogeneity may also be influenced by other factors, including measured factors such as the length of the study period, study design, and laboratory technique. For example, while most studies used highly sensitive and specific diagnostic PCR tests to detect these viruses, studies which used less sensitive diagnostic methods likely underestimated prevalence. Heterogeneity may also have been influenced by unmeasured factors. Selection of participants may also have been different among the studies, likely resulting in higher prevalence among studies in which patients with more severe illness were enrolled, such as SARI as compared to ILI patients. Additionally, the sample size of studies eligible for inclusion in this analysis was small, limiting the power to detect differences, particularly in subgroup analysis. For example, not all countries of the EAC were represented, and Tanzania and Uganda only had one study each. The small sample size may be due to various factors including limited funding, government policy and priorities, the challenges of information sharing, lack of documentation, inaccessibility of databases, and difficulties with publication of data. Further, the presence of extreme values of prevalence introduced computational complexity which limited our ability to report confidence intervals for the I^2^ values.

Finally, this review only included studies in which patients were selected based on defined medical conditions such as ILI and SARI or ARTIs in general. Studies that included asymptomatic participants were excluded. Asymptomatic patients would likely have had lower prevalence of these viruses than the symptomatic populations used in our study. Therefore the results of this review cannot be generalized to the general population. The exclusion of asymptomatic cases was considered necessary to avoid overdiagnosis of patients who were colonized or carriers of viruses which may not have ever caused disease.

Despite these limitations of the study, this study had several strengths. We have documented the first systematic review and use of meta-analysis to estimate the pooled prevalence of selected non-influenza viruses in the EAC. This systematic review and meta-analysis simultaneously reported HRSV, HPIV, and HAdV prevalence with a pre-defined protocol, used robust search strategies, and involved two independent investigators. Selected studies were all assessed for well-defined study characteristics to assess study heterogeneity and bias. There was no significant publication bias detected, and most of the included studies had a low risk of study bias. Sensitivity analysis yielded similar results to crude estimates, further supporting the robustness of this systematic review and meta-analysis.

## Conclusions and recommendations

Respiratory illness surveillance programs in the EAC have enhanced the detection of both influenza and non-influenza viruses for over a decade. However, there are no platforms for systematic information sharing in the region. It is vital to establish national and regional information-sharing platforms for non-influenza respiratory viruses to guide future research, policy, and development. Our findings indicate that human adenoviruses are the most common sources of ILI and SARI other than influenza infection, followed by the human respiratory syncytial virus and parainfluenza virus. Future studies or research could identify the prevalence of HRSV, HPIV, and HAdV using standardized methods and populations to increase comparability among studies and to account for sources of misclassification and heterogeneity. Additional studies should be considered among older populations, among populations from EAC countries from which no data were found, and asymptomatic populations. In addition, the literature search could include additional databases used in biomedical research. Finally, other emerging respiratory pathogens could be studied and further molecular characterization could be carried out to assess transmission.

## Supporting information

S1 File Search strategies. (PDF)

S2 File PRISMA checklist. (PDF)

S3 File List of individual study characteristics. (TIF)

S4 File Individual study bias assessment. (PDF)
