# Supplementary material for: Prevalence of human respiratory syncytial virus, parainfluenza and adenoviruses in East Africa Community partner states of Kenya, Tanzania, and Uganda: A systematic review and meta-analysis (2007–2020)
Source: PLoS One. 2021 Apr 27;16(4):e0249992. doi: 10.1371/journal.pone.0249992 (PMC8078816; doi:10.1371/journal.pone.0249992)
Supplement: S1 File — (PDF) [file pone.0249992.s001.pdf]

Appendices Search strategies

a) Search strategy PubMed database

Search number	Query
21	((((((prevalence[Title/Abstract]) OR ("Prevalence"[Mesh] OR "epidemiology" [Subheading] OR "Cross-Sectional Studies"[Mesh])) OR incidence[Title/Abstract]) OR ("Incidence"[Mesh] OR "epidemiology" [Subheading] OR "Cohort Studies"[Mesh]))) AND ((((((((((((acute viral respiratory infection[Title/Abstract]) OR influenza like illness[Title/Abstract]) OR severe acute respiratory illness[Title/Abstract]) OR respiratory syncytial virus[Title/Abstract]) OR ("Respiratory Syncytial Viruses"[Mesh] OR "Respiratory Syncytial Virus Infections"[Mesh] OR "Respiratory Syncytial Virus, Human"[Mesh])) OR parainfluenza virus[Title/Abstract]) OR ("Parainfluenza Virus 5"[Mesh] OR "Parainfluenza Virus 4, Human"[Mesh] OR "Parainfluenza Virus 3, Human"[Mesh] OR "Parainfluenza Virus 2, Human"[Mesh] OR "Parainfluenza Virus 1, Human"[Mesh])) OR adenovirus[Title/Abstract]) OR ("Adenoviridae Infections"[Mesh] OR "Adenovirus Infections, Human"[Mesh] OR "Adenoviruses, Human"[Mesh])) OR other respiratory virus[Title/Abstract]) OR pneumonia[Title/Abstract]) OR ("Pneumonia"[Mesh] OR "Pneumonia, Viral"[Mesh]))) AND ((east african community[Title/Abstract]) OR ((((((Rwanda[Title/Abstract]) OR Burundi) OR Uganda) OR Kenya) OR Tanzania) OR South Sudan)) Filters: from 2007/1/1 - 2020/12/31
20	Search (east african community[Title/Abstract]) OR ((((((Rwanda[Title/Abstract]) OR Burundi) OR Uganda) OR Kenya) OR Tanzania) OR South Sudan)
19	Search ((((((((((((acute viral respiratory infection[Title/Abstract]) OR influenza like illness[Title/Abstract]) OR severe acute respiratory illness[Title/Abstract]) OR respiratory syncytial virus[Title/Abstract]) OR ("Respiratory Syncytial Viruses"[Mesh] OR "Respiratory Syncytial Virus Infections"[Mesh] OR "Respiratory Syncytial Virus, Human"[Mesh])) OR parainfluenza virus[Title/Abstract]) OR ("Parainfluenza Virus 5"[Mesh] OR "Parainfluenza Virus 4, Human"[Mesh] OR "Parainfluenza Virus 3, Human"[Mesh] OR "Parainfluenza Virus 2, Human"[Mesh] OR "Parainfluenza Virus 1, Human"[Mesh])) OR adenovirus[Title/Abstract]) OR ("Adenoviridae Infections"[Mesh] OR "Adenovirus Infections, Human"[Mesh] OR "Adenoviruses, Human"[Mesh])) OR other respiratory virus[Title/Abstract]) OR pneumonia[Title/Abstract]) OR ("Pneumonia"[Mesh] OR "Pneumonia, Viral"[Mesh])
18	Search (((prevalence[Title/Abstract]) OR ("Prevalence"[Mesh] OR "epidemiology" [Subheading] OR "Cross-Sectional Studies"[Mesh])) OR incidence[Title/Abstract]) OR ("Incidence"[Mesh] OR "epidemiology" [Subheading] OR "Cohort Studies"[Mesh])
17	Search (((((Rwanda[Title/Abstract]) OR Burundi) OR Uganda) OR Kenya) OR Tanzania) OR South Sudan
16	Search east african community[Title/Abstract]
15	Search "Pneumonia"[Mesh] OR "Pneumonia, Viral"[Mesh]
14	Search pneumonia[Title/Abstract]
13	Search other respiratory virus[Title/Abstract]
12	Search "Adenoviridae Infections"[Mesh] OR "Adenovirus Infections, Human"[Mesh] OR "Adenoviruses, Human"[Mesh]
11	Search adenovirus[Title/Abstract]
10	Search "Parainfluenza Virus 5"[Mesh] OR "Parainfluenza Virus 4, Human"[Mesh] OR "Parainfluenza Virus 3, Human"[Mesh] OR "Parainfluenza Virus 2, Human"[Mesh] OR "Parainfluenza Virus 1, Human"[Mesh]

9 Search parainfluenza virus[Title/Abstract]
8 Search "Respiratory Syncytial Viruses"[Mesh] OR "Respiratory Syncytial Virus Infections"[Mesh] OR "Respiratory Syncytial Virus, Human"[Mesh]
7 Search severe acute respiratory illness[Title/Abstract]
6 Search influenza like illness[Title/Abstract]
5 Search acute viral respiratory infection[Title/Abstract]
4 Search "Incidence"[Mesh] OR "epidemiology" [Subheading] OR "Cohort Studies"[Mesh]
3 Search incidence[Title/Abstract]
2 Search "Prevalence"[Mesh] OR "epidemiology" [Subheading] OR "Cross-Sectional Studies"[Mesh]
1 Search prevalence[Title/Abstract]

b) Search strategy Global Index Medicus database

tw:((tw:(prevalence)) OR (tw:(cross-sectional studies)) OR (tw:(meta-analysis)) OR (tw:(incidence)) OR (tw:(cohort studies)) OR (tw:(epidemiology studies)) AND (tw:(respiratory tract infections)) OR (tw:(upper respiratory tract infections)) OR (tw:(pneumonia, viral)) OR (tw:(respiratory syncytial virus, human)) OR (tw:(parainfluenza virus 1, human)) OR (tw:(parainfluenza virus 2, human)) OR (tw:(parainfluenza virus 3, human)) OR (tw:(adenovirus infections, human)) AND (tw:(africa, eastern)) OR (tw:(kenya)) OR (tw:(tanzania)) OR (tw:(uganda)) OR (tw:(rwanda)) OR (tw:(burundi)) OR (tw:(south sudan))) AND (la:("en")) AND (year_cluster:[2007 TO 2020])
