# Supplementary material for: Prevalence of human respiratory syncytial virus, parainfluenza and adenoviruses in East Africa Community partner states of Kenya, Tanzania, and Uganda: A systematic review and meta-analysis (2007–2020)
Source: PLoS One. 2021 Apr 27;16(4):e0249992. doi: 10.1371/journal.pone.0249992 (PMC8078816; doi:10.1371/journal.pone.0249992)
Supplement: S2 File — (PDF) [file pone.0249992.s002.pdf]

PRISMA 2009 Checklist

Section/topic	#	Checklist item	Reported on page #
**TITLE**			
Title	1	Prevalence of human respiratory syncytial virus, parainfluenza and adenoviruses in East Africa Community partner states of Kenya, Tanzania, and Uganda: a systematic review and meta-analysis (2007-2020)	Page 1
**ABSTRACT**			
Structured summary	2	**Background**<br>Viruses are responsible for a large proportion of acute respiratory tract infections (ARTIs). Human influenza, parainfluenza, respiratory-syncytial-virus, and adenoviruses are among the leading cause of ARTIs. Epidemiological evidence of those respiratory viruses is limited in the East Africa Community (EAC) region. This review sought to identify the prevalence of respiratory syncytial virus, parainfluenza, and adenoviruses among cases of ARTI in the EAC from 2007 to 2020<br>**Methods**<br>A literature search was conducted in Medline, Global Index Medicus, and the grey literature from public health institutions and programs in the EAC. Two independent reviewers performed data extraction. We used a random effects model to pool the prevalence estimate across studies. We assessed heterogeneity with the I2 statistic, and Cochran's Q test, and further we did subgroup analysis. This review was registered with PROSPERO under registration number CRD42018110186.<br>**Results**<br>A total of 12 studies met the eligibility criteria for the studies documented from 2007 to 2020. The overall pooled prevalence of adenoviruses was 13% (95% confidence interval [CI]: 6-21, N=28829), respiratory syncytial virus 11% (95% CI: 7-15, N=22627), and parainfluenza was 9% (95% CI: 7-11, N= 28363). Pooled prevalence of reported ARTIs, all ages, and locality varied in the included studies. Studies among participants with severe acute respiratory disease had a higher pooled prevalence of all the three viruses. Considerable heterogeneity was noted overall and in subgroup analysis.<br>**Conclusion**<br>Our findings indicate that human adenoviruses, respiratory syncytial virus and parainfluenza virus are prevalent in Kenya, Tanzania, and Uganda. These three respiratory viruses contribute substantially to ARTIs in the EAC, particularly among those with severe disease and those aged five and above.	Page 2-3
**INTRODUCTION**			
Rationale	3	Acute respiratory tract infections (ARTIs) are among the five most common causes of morbidity and mortality globally, accounting for approximately 3.9 million deaths annually. Most of these deaths occur among young children in developing countries [1]. Viruses are responsible for a large proportion of ARTIs and these are associated with various syndromes of the upper and lower respiratory tract, including: acute otitis media, croup, pneumonia, bronchiolitis, and asthma [2,3]. Additionally, co-infections of viruses and bacteria are commonly reported in severe cases of ARTIs [4,5]. Although viral aetiologies are associated with a large percentage of acute respiratory tract infections, it is difficult to link specific viral agents to a specific syndrome. This is due to the complexity and broad spectrum of illnesses caused by these pathogens. Furthermore, the emergence of new viral strains and cost of	Page 4-6

		diagnosis contribute to the inability to detect viral agents in order to associate these pathogens with a specific syndrome. However, influenza viruses, one of the major causative agents of acute respiratory tract infections, have been extensively studied with an established global surveillance program. This program was set up to assess the threat of the emergence of strains which could cause pandemic disease [6].<br><br>Globally, non-influenza respiratory viruses have received less attention respiratory virus surveillance programs, hence few studies are available in the published literature [7]. Nevertheless, a few previous studies have indicated the risk of non-influenza viruses to public health, and that some viral families have the potential to cause epidemics [8]. The non-influenza respiratory viruses most commonly associated with ARTIs include human respiratory syncytial viruses (HRSV), parainfluenza viruses (HPIVs), and adenoviruses (HAdV), among others [9]. The consequences of these respiratory viruses result in an enormous direct and indirect economic burden on public health. In the United States alone, the estimated annual economic burden of non-influenza viral respiratory tract infections is equivalent to $40 billion [10]. Interestingly, a global incidence of at least 33.1 million has been associated with HRSV in young children under five [11]. The same study indicated a mortality range of 48,000–74,500 for children younger than 5 years and estimated that 99% of these deaths occurred in developing countries [11].<br><br>In Sub-Saharan Africa, recent annual incidence data of community-acquired pneumonia is estimated to be 131 million, with significant proportion of these aetiologies due to viruses [12]. A study conducted in Senegal reported that a range of respiratory viruses cause influenza-like illness (ILI) with substantial proportions due to influenza viruses (53.1%; 1045/1967), rhinoviruses (30%; 591/1967), enteroviruses (18.5%; 364/1967), and HRSV (13.5%; 266/1967) in children under five years old [13]. A review of the aetiology of ARTIs in children <5 years in Sub-Saharan Africa showed that HRSVs, HPIVs, and HAdV were among the leading causes of ARTIs [14]. Moreover, in 2018 a systematic review and meta-analysis of HRSV prevalence in Africa reported an overall HRSV prevalence of 14%, thus indicating that this pathogen contributes significantly to severe respiratory illness on the continent [15].<br><br>The World Health Organization Regional Office for Africa (WHO-AFRO) 2012 country profiles indicated that acute lower respiratory infections (ALRTIs) were amongst the top three causes of death in the East African Community (EAC). In EAC partner states, the proportionate mortality from lower respiratory tract infections (LRTIs) was: Tanzania (8.7%), Kenya (12.3%), Uganda (9.6%), South Sudan (12%), Rwanda (10%) and Burundi (12.5%). Amongst these EAC states, Kenya, Tanzania, Uganda, and Rwanda have established surveillance programs for influenza and other respiratory viruses which are recognized by the WHO [16]. The EAC has a large population with approximately 161 million inhabitants [17] who are highly mobile and integrated, with a common regional market, tourism, and social and cultural exchange. These factors increase the risk of infectious disease transmission and spread in the EAC, as has been described elsewhere [18]. Several other studies have demonstrated various viral aetiologies as causes of ARTIs in the EAC [19,20]. In Kenya, a study conducted at the Kilifi district hospital reported a high prevalence (34%) of HRSV infections in young children which was associated with severe pneumonia [21]. Human parainfluenza, adenoviruses, and other respiratory viruses were also reported [21]. The use of molecular techniques have also enhanced the detection and identification of other non-influenza respiratory viruses in countries with no consistent respiratory disease surveillance programs [22].<br><br>A large number of programs for surveillance of influenza-like illness (ILI) and severe acute respiratory illness (SARI) were established in order to strengthen health security after the establishment of the 2005 International Health Regulations. However, such programs have primarily estimated the occurrence of influenza viruses and have either not assessed or not reported other (non-influenza) viruses [23]. This leaves an inconsistent assessment of the epidemiology of non-influenza viruses in the EAC region. This review addresses some of this gap by providing a systematic review of the published and unpublished literature of pooled prevalence of HRSV, HPIV, and HAdV	

		among symptomatic patients in EAC partner states over the period between 2007 and 2020. These three viruses were the most frequent non-influenza respiratory viruses detected in the surveillance programs; the prevalence of other non-influenza viruses was rarely reported, precluding formal systematic review.	
Objectives	4	This review addresses the questions or the gaps of pooled prevalence of HRSV, HPIV, and HAdV among symptomatic patients in EAC partner states over study period from 2007 to 2020 by providing a systematic review of the published and unpublished literature.	Page 6
**METHODS**			
Protocol and registration	5	PROSPERO: CRD42018110186	Page 10
Eligibility criteria	6	This review considered studies that reported laboratory-confirmed infections caused by HRSV, HPIV, and HAdV in all age groups. These three respiratory viruses are among those frequently reported to cause respiratory tract infections other than influenza. In addition, the review included a broad range of study participants, including those with acute respiratory tract infections (ARTIs), influenza-like illnesses (ILIs), severe acute respiratory illnesses (SARIs), and other syndromes including pneumonia. Studies reporting asymptomatic infections were excluded in this review.<br><br>The review considered observational studies, including prospective and retrospective cross-sectional and cohort studies that were either descriptive, analytical, or both. Case series, individual case reports, letters to editors, reviews, commentaries, and qualitative studies were excluded. Only studies published in English, including those from unpublished reports from the grey literature, were included. Published studies and unpublished reports documented in the period between 1st January 2007 and 31st December 2020 were included.	Page 7
Information sources	7	An initial unlimited search was conducted in Medline that allowed more refined search strategies tailored for Global Index Medicus. The initial search was performed by verification of the text words contained in the title and abstract of the index terms, which were used to describe the articles using keywords and Medical Subject Heading (MeSH) terminologies (S1). This informed the development of a search strategy that was used for each information source. In addition, reference lists of all studies selected for inclusion were screened for additional relevant publications. The search was first completed in 2019 then updated using the same methodology in 2021.<br><br>To obtain information from the grey literature, inquiries regarding these viruses were made directly to the ministries of health. We also searched the databases of government medical research institutions, teaching hospitals, and university libraries in EAC partner states. Electronic database search or author correspondence was performed with the Kenya Medical Research Institute (KEMRI) and Kenyatta National Hospital (KNH) for Kenya; National Institute for Medical Research (NIMR) for Tanzania); Uganda Virus Research Institute (UVRI) and Makerere University (MAK), and Mulago Hospital (MUH) for Uganda; Institut National de Sante Public (INSP) for Burundi; and Rwanda Biomedical Center (RBC) for Rwanda. In addition, other non-government public health research programs in the East African Community were contacted.<br><br>All identified citations were collated and uploaded into Zotero software, version 5.0, (Corporation for Digital Scholarship, Vienna, VA) and duplicates were removed. Titles and abstracts were re-screened against the eligibility criteria, and studies that met the eligibility criteria were retrieved in full and their details imported into JBI SUMARI (Joanna Briggs Institute, Adelaide, Australia). The full texts of selected studies were assessed in detail against the eligibility criteria by two parallel reviewers, and any disagreements were resolved by a third investigator.	Page 7-8
Search	8	Search number Query<br><br>21 ((((((prevalence[Title/Abstract]) OR ("Prevalence"[Mesh] OR "epidemiology" [Subheading] OR "Cross-	Page 25

		Sectional Studies"[Mesh])) OR incidence[Title/Abstract]) OR ("Incidence"[Mesh] OR "epidemiology" [Subheading] OR "Cohort Studies"[Mesh]))) AND ((((((((((((acute viral respiratory infection[Title/Abstract]) OR influenza like illness[Title/Abstract]) OR severe acute respiratory illness[Title/Abstract]) OR respiratory syncytial virus[Title/Abstract]) OR ("Respiratory Syncytial Viruses"[Mesh] OR "Respiratory Syncytial Virus Infections"[Mesh] OR "Respiratory Syncytial Virus, Human"[Mesh])) OR parainfluenza virus[Title/Abstract]) OR ("Parainfluenza Virus 5"[Mesh] OR "Parainfluenza Virus 4, Human"[Mesh] OR "Parainfluenza Virus 3, Human"[Mesh] OR "Parainfluenza Virus 2, Human"[Mesh] OR "Parainfluenza Virus 1, Human"[Mesh])) OR adenovirus[Title/Abstract]) OR ("Adenoviridae Infections"[Mesh] OR "Adenovirus Infections, Human"[Mesh] OR "Adenoviruses, Human"[Mesh])) OR other respiratory virus[Title/Abstract]) OR pneumonia[Title/Abstract]) OR ("Pneumonia"[Mesh] OR "Pneumonia, Viral"[Mesh]))) AND ((east african community[Title/Abstract]) OR ((((((Rwanda[Title/Abstract]) OR Burundi) OR Uganda) OR Kenya) OR Tanzania) OR South Sudan)) Filters: from 2007/1/1 - 2020/12/31<br>20 Search (east african community[Title/Abstract]) OR ((((((Rwanda[Title/Abstract]) OR Burundi) OR Uganda) OR Kenya) OR Tanzania) OR South Sudan)<br>19 Search ((((((((((acute viral respiratory infection[Title/Abstract]) OR influenza like illness[Title/Abstract]) OR severe acute respiratory illness[Title/Abstract]) OR respiratory syncytial virus[Title/Abstract]) OR ("Respiratory Syncytial Viruses"[Mesh] OR "Respiratory Syncytial Virus Infections"[Mesh] OR "Respiratory Syncytial Virus, Human"[Mesh])) OR parainfluenza virus[Title/Abstract]) OR ("Parainfluenza Virus 5"[Mesh] OR "Parainfluenza Virus 4, Human"[Mesh] OR "Parainfluenza Virus 3, Human"[Mesh] OR "Parainfluenza Virus 2, Human"[Mesh] OR "Parainfluenza Virus 1, Human"[Mesh])) OR adenovirus[Title/Abstract]) OR ("Adenoviridae Infections"[Mesh] OR "Adenovirus Infections, Human"[Mesh] OR "Adenoviruses, Human"[Mesh])) OR other respiratory virus[Title/Abstract]) OR pneumonia[Title/Abstract]) OR ("Pneumonia"[Mesh] OR "Pneumonia, Viral"[Mesh])<br>18 Search (((prevalence[Title/Abstract]) OR ("Prevalence"[Mesh] OR "epidemiology" [Subheading] OR "Cross-Sectional Studies"[Mesh])) OR incidence[Title/Abstract]) OR ("Incidence"[Mesh] OR "epidemiology" [Subheading] OR "Cohort Studies"[Mesh])<br>17 Search (((((Rwanda[Title/Abstract]) OR Burundi) OR Uganda) OR Kenya) OR Tanzania) OR South Sudan<br>16 Search east african community[Title/Abstract]<br>15 Search "Pneumonia"[Mesh] OR "Pneumonia, Viral"[Mesh]<br>14 Search pneumonia[Title/Abstract]<br>13 Search other respiratory virus[Title/Abstract]<br>12 Search "Adenoviridae Infections"[Mesh] OR "Adenovirus Infections, Human"[Mesh] OR "Adenoviruses, Human"[Mesh]<br>11 Search adenovirus[Title/Abstract]<br>10 Search "Parainfluenza Virus 5"[Mesh] OR "Parainfluenza Virus 4, Human"[Mesh] OR "Parainfluenza Virus 3, Human"[Mesh] OR "Parainfluenza Virus 2, Human"[Mesh] OR "Parainfluenza Virus 1, Human"[Mesh]<br>9 Search parainfluenza virus[Title/Abstract]<br>8 Search "Respiratory Syncytial Viruses"[Mesh] OR "Respiratory Syncytial Virus Infections"[Mesh] OR "Respiratory Syncytial Virus, Human"[Mesh]<br>7 Search severe acute respiratory illness[Title/Abstract]<br>6 Search influenza like illness[Title/Abstract]	

PRISMA 2009 Checklist

		5 Search acute viral respiratory infection[Title/Abstract]<br>4 Search "Incidence"[Mesh] OR "epidemiology" [Subheading] OR "Cohort Studies"[Mesh]<br>3 Search incidence[Title/Abstract]<br>2 Search "Prevalence"[Mesh] OR "epidemiology" [Subheading] OR "Cross-Sectional Studies"[Mesh]<br>1 Search prevalence[Title/Abstract]	
Study selection	9	Before data extraction, all selected studies were critically appraised for methodological quality. This was accomplished with a standardized critical appraisal instrument from the Joanna Briggs Institute by two independent reviewers. This review followed the guideline of systematic reviews of prevalence and incidence manual with the use of JBI SUMARI software available at //www.jbisumari.org.	Page 8
Data collection process	10	All data extracted from selected studies were included in the review using a standardized data extraction tool in JBI SUMARI software by the two independent reviewers.	Page 8
Data items	11	Extracted data consisted of specific details about the names of the primary author, year of publication, locality, age category, clinical condition, study design, length of the study period, specimen type, laboratory test, number of cases, and total population.	Page 8
Risk of bias in individual studies	12	The risk of bias was assessed in the selected studies through the use of an eight variable rating scale [24–27]. Each was given a score for how well-defined and clearly reported the variable was. The variables included: i) length of a study period which was at least three months to permit laboratory processing of samples and analysis, ii) year of documentation or publication, iii) study area (country or study locality), iv) age group description, v) clinical condition or syndrome using a standard case definition, vi) standardized type of specimen collection, vii) laboratory methodology and viii) type of study design. Each variable received a score of one for a clear and defined record and zero for missing or unclear documentation. Thus, the scores could range from 0 to 8. We categorized scores of 0 to 2 as high risk for bias, 3 to 5 as medium risk, and 6 to 8 as low risk.	Page 9
Summary measures	13	Prevalence (proportion)	Page 9
Synthesis of results	14	Data were analysed using Stata®13 (StataCorp, College Station, TX). The dataset was re-organized and coded for analysis, and further meta-analysis was performed using the “metaprop” package in STATA program [28]. Initially, unadjusted prevalence of HRSV, HPIV and HAdV infections were calculated based on the crude numerators and denominators found among the individual studies.<br>To ensure that studies with very small or large prevalence were kept in the overall estimates, the Freeman-Tukey double-arcsine transformation technique was performed using the metaprop command [29]. This procedure stabilized the variance of study-specific prevalence before applying a random-effects model (RE) to assess heterogeneity and generate a pooled prevalence estimate. The random-effects model allowed the effect to vary across studies, providing more conservative estimates with wider confidence intervals given the observed heterogeneity between studies [30]. It was implemented using the method of DerSimonian and Laird [31], whereas 95% confidence intervals (CIs) were drawn from exact binomial distribution (Clopper-Pearson) [32]. The I2 statistic, Cochran’s Q test, and subgroup analysis were used to assess heterogeneity [33]. The statistical values of I2 expressed the variation of in-between studies differences as a percentage, simplifying the interpretation with the “rule of thumb” [33, 34]. Generally, a substantial heterogeneity was indicated by the values of I2 >50%, whereas a tentative categories of minimal (I2<=25%), low (I2=25-4950%), moderate (I2=50-75%), and high (I2>=75%) [33, 35].	Page9-10

PRISMA 2009 Checklist

Section/topic	#	Checklist item	Reported on page #
Risk of bias across studies	15	Further assessment, a funnel plot was generated, and an egger test was performed with a metabias command to evaluate the publication bias refer to the “small-study effects” [36]. The Egger test of P<0.10 indicated a significant publication bias [37–39].	Page9-10
Additional analyses	16	Prevalence of infection was described by country, age group, and clinical conditions. In addition, pie-charts were used to display the prevalence of HRSV, HPIV and HAdV infections in the region with quantum geographical information system (qGIS). Subgroup analyses were performed on variables of public health importance including; clinical condition (ILI, SARI, or ARTIs), age groups (below five, five and above, or all ages) and locality (Kenya, Tanzania, or Uganda). We followed guidelines for systematic reviews of prevalence and incidence from the Joanna Briggs Institute to accomplish this review. In addition, the Preferred Reporting Items for Systematic Reviews and Meta-Analyses (PRISMA) guided the report writing (S2). This review was registered in the International Prospective Register of Systematic Reviews (PROSPERO) under registration number CRD42018110186.	Page9-10
**RESULTS**			
Study selection	17	In this review, we found 1005 records in published databases and 5 reports from unpublished sources. After filtering based on the defined study period (2007-2020), 995 (990 published and 5 unpublished) studies were retained. A total of 299 (294 published and 5 unpublished) were retained after removing 188 duplicates. After screening using the abstract and title, we excluded 508 which did not meet eligibility criteria. The remaining 26 studies were assessed for full article eligibility, and 14 were excluded for different reasons shown in Figure 1. Twelve (12) studies met eligibility criteria and were therefore included in the study with further meta-analysis.	Page10

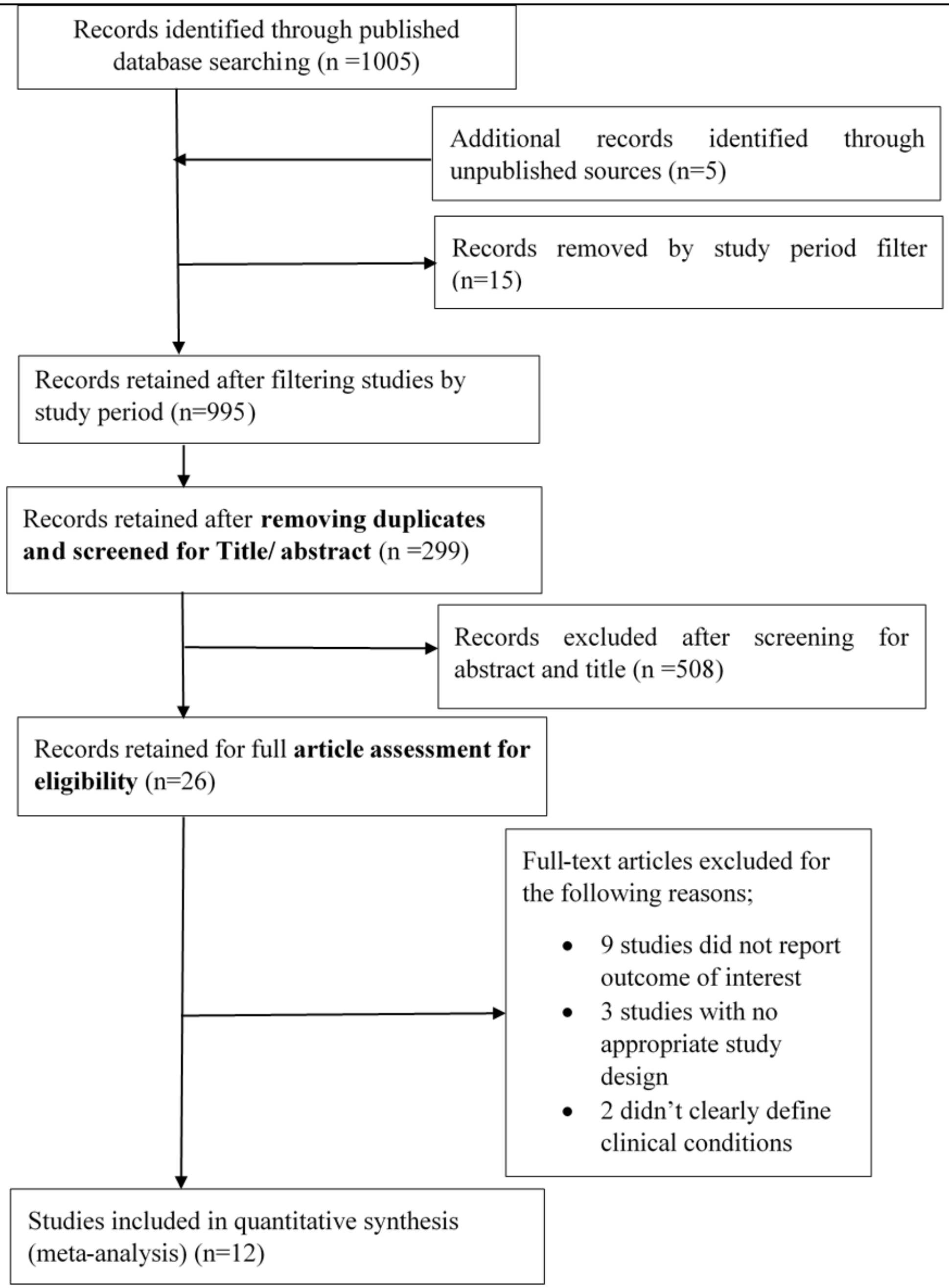
Records identified through published database searching (n =1005)
Additional records identified through unpublished sources (n=5)
Records removed by study period filter (n=15)
Records retained after filtering studies by study period (n=995)
Records retained after removing duplicates and screened for Title/ abstract (n =299)
Records excluded after screening for abstract and title (n =508)
Records retained for full article assessment for eligibility (n=26)
Full-text articles excluded for the following reasons;
• 9 studies did not report outcome of interest
• 3 studies with no appropriate study design
• 2 didn't clearly define clinical conditions
Studies included in quantitative synthesis (meta-analysis) (n=12)

Study characteristics	18	In this review, qualified studies were documented from 2009 to 2018 (S3). A large number of studies on the selected viruses were conducted in Kenya (Table 1). These comprised studies of HRSV (n=8, 80%), HPIV (n=6, 75%) and HAdV (n=7, 77.7%) (S4). Tanzania and Uganda had one study each which reported results for all three of the viruses under investigation. There were no available studies from Rwanda, Burundi, and South Sudan that assessed any of the three viruses during the period under review. Most studies reported for the three countries were cross-sectional. Furthermore, the majority of studies (n=6) were of acute respiratory tract infections (ARTIs) involving both ILI and SARI. A few studies (n=4) reported ILI only, and 2 were SARI only. Five studies reported having enrolled individuals of all ages, while four studies recruited participants aged under five years only, and one study exclusively enrolled participants aged five years and older. The majority (n=7) of studies were conducted over a period of >12 months, two were <5 months, and one was 6-12 months. Polymerase chain reaction (PCR) was the most common diagnostic test, and most studies collected and analysed both oropharyngeal swabs (OPS) and nasopharyngeal swabs (NPS) specimens [1–12].<br>1. Jamal A. Ahmed, Mark A. Katz, Eric Auko, M. Kariuki Njenga, Michelle Weinberg, Bryan K. Kapella, et al. Epidemiology of respiratory viral infections in two long-term refugee camps in Kenya, 2007-2010. BMC Infect Dis. 2012 Jan 17;12:7.<br>2. Gedi A. Mohamed, Jamal A. Ahmed, Nina Marano, Abdinoor Mohamed, Edna Moturi, Wagacha Burton, et al. Etiology and Incidence of Viral Acute Respiratory Infections Among Refugees Aged 5 Years and Older in Hagadera Camp, Dadaab, Kenya. Am J Trop Med Hyg. 2015 Dec;93(6):1371–6.<br>3. Rosemarie Gachie- Lopokoiyit. Prevalence of respiratory viral pathogens in nasopharyngeal and oropharyngeal specimens and clinical outcomes in young children presenting with severe acute respiratory infections at Kenyatta National Hospital [Internet] [Thesis]. University of Nairobi; 2014 [cited 2018 Oct 2]. Available from: http://erepository.uonbi.ac.ke:8080/xmlui/handle/11295/76601<br>4. S. M. I. Symekhier, W. O. Ochieng, J. Simwa. Prevalence of viral aetiologies in children with acute respiratory infections in Nairobi, Kenya. Tanzania Journal of Health Research [Internet]. 2009 Jan 1 [cited 2018 Sep 28];11(2). Available from: https://www.ajol.info/index.php/thrb/article/view/45210<br>5. Rachel A. Achilla, Wallace D. Bulimo, Janet M. Majanja, Meshack O. Wadegu, Silvanus O. Mukunzi, Josphat Mwangi, et al. Respiratory Adenovirus Species Circulating In Kenya from 2007-2010. African Journal of Pharmacology and Therapeutics [Internet]. 2012 Dec 31 [cited 2017 Dec 25];1(4). Available from: http://journals.uonbi.ac.ke/ajpt/article/view/1110<br>6. Keneth Mitei, Wallace Bulimo, Rachel Achilla, Janet Majanja, Meshack Wadegu. Surveillance of Human Parainfluenza viruses in Kenya during the 2007-2011 Period. African Journal of Pharmacology and Therapeutics [Internet]. 2012 Dec 31 [cited 2017 Dec 24];1(4). Available from: http://journals.uonbi.ac.ke/ajpt/article/view/1118<br>7. Joyce Uchi Nyiro, Patrick Munywoki, Everlyn Kamau, Charles Agoti, Alex Gichuki, Timothy Etyang, et al. Surveillance of respiratory viruses in the outpatient setting in rural coastal Kenya: baseline epidemiological observations. Wellcome Open Res. 2018;3:89.<br>8. Nyawanda Bryan O, Otieno Nancy A., Omollo Daniel, Otieno Micheal, Oneko Martina, Bigogo Godfrey, et al.	Page 11

		The Burden of Influenza and Respiratory Syncytial Virus in Infants 0-2 Months Old in Rural Western Kenya; Preliminary Data, 2015-2017. In 2018. Available from: http://www.anise-network.org/<br><br>9. Gideon O. Emukule, Sammy Khagayi, Meredith L. McMorrow, Rachel Ochola, Nancy Otieno, Marc-Alain Widdowson, et al. The burden of influenza and RSV among inpatients and outpatients in rural western Kenya, 2009-2012. PLoS ONE. 2014;9(8):e105543.<br><br>10. Mmbaga Vida, Maria Kelly, Matonya Miriam, Mwalongo Vumilia, Mosha Fausta, Moshi Solomoni, et al. The Prevalence of Influenza and other Respiratory Viruses among ILI and SARI patients in Tanzania, from January 2016 to August 2017. In Carlton Hotel Antananarivo, Madagascar; 2018. Available from: http://www.anise-network.org/<br><br>11. Stephen Balinandi, Barnabas Bakamutumaho, John T. Kayiwa, Juliette Ongus, Joseph Oundo, Anna C. Awor, et al. The viral aetiology of influenza-like illnesses in Kampala and Entebbe, Uganda, 2008. Afr J Lab Med. 2013 Jun 24 ;2(1). Available from: https://www.ncbi.nlm.nih.gov/pmc/articles/PMC5637772/<br><br>12. Daniel R. Feikin, M. Kariuki Njenga, Godfrey Bigogo, Barrack Aura, George Aol, Allan Audi, et al. Viral and Bacterial Causes of Severe Acute Respiratory Illness Among Children Aged Less Than 5 Years in a High Malaria Prevalence Area of Western Kenya, 2007–2010. The Pediatric Infectious Disease Journal. 2013 Jan;32(1):e14.	
Risk of bias within studies	19	It was noted that in general, studies had a low risk of bias. Studies reported minimal bias for the HRSV (90%), HPIV (87.5%), and HAdV (88.8%)(Table 1).	Page 11
Results of individual studies	20	The overall pooled prevalence of HRSV was 11% (7-15; 95% CI) reported from 10 studies with a total population of 22,627 participants (S1 Fig). The estimated overall pooled prevalence of 9% (7-11; 95% CI) HPIV was estimated from 8 studies with 28,363 participants (S2 Fig). HAdV overall pooled prevalence was 13% (6-21; 95% CI) recorded in 9 studies with 28,829 participants (S3 Fig)	Page12-13
Synthesis of results	21	A pooled prevalence of 10% (6-15; 95% CI) was found for HRSV, 9% (7-11; 95% CI) for HPIV, and 12% (3-27; 95% CI) for HAdV when restricting the analysis to the studies that investigated ARTIs. Prevalence estimates from ILI studies only found HRSV, HPIV and HAdV prevalences of 5% (95% CI: 4-7, 5% (95% CI: 5-6), and 3% (95% CI: 3-3.5) respectively. In contrast, estimates from SARI studies only were higher for all three viral pathogens at 22% (95% CI: 20-23) for HRSV, 16% (95% CI: 12-21) for HPIV and 18% (95% CI: 16-19) for HAdV.<br><br>Studies which considered participants of all ages had an estimated prevalence of 9% (95% CI: 5-14), 9% (95% CI: 6-12) and 12% (95% CI: 4-24) for HRSV, HPIV, and HAdV, respectively. Prevalences of 10% (95% CI: 2-24) for HRSV, 6% (95% CI: 4-9) for HPIV, and 15% (95% CI: 13-16) for HAdV were reported in studies that enrolled participants who were under five years of age. In the studies that involved individuals five years and above, HSRV prevalence was similar at 10% (95% CI: 6-15), whereas the prevalence of HPIV and HAdV was much higher at 14% (95% CI: 9-21) and 30% (95% CI: 23-37) respectively.<br><br>For studies carried out in Kenya, prevalences of HRSV, HPIV and HAdV were 10% (95% CI: 6-15), 9% (95% CI: 7-11) and 14% (95% CI: 7-25) respectively. In Tanzania, estimated prevalence of HAdV and HPIV were similar at 9% (95% CI: 6-12) and 10% (95% CI: 7-14), whereas HRSV prevalence was higher at 29% (95% CI: 24-35). In	Page 13

		the studies conducted in Uganda, the corresponding prevalences of HRSV, HPIV, and HAdV were lower at 3% (95% CI: 2-6), 6% (95% CI: 4-9), and 8% (95% CI: 5-12).	
Risk of bias across studies	22	There was no publication bias suggested by the funnel plot and or the Egger's test (Table 2). Whereas considerable heterogeneity was noted overall and in subgroup analysis, no publication bias was recorded in analysis of subgroups with enough studies to assess (ARTIs, All ages, and Kenya).	Page 14
Additional analysis	23	A similar prevalence to the overall prevalence was documented when restricting the analysis to studies of ARTI: 12% (95% CI: 3-27) for HAdV, 10% (95% CI: 6-15) for HRSV, and 9% (95% CI: 7-11) for HPIV. In addition, studies that involved participants of all ages had a pooled prevalence of 12% (95% CI: 4-24) for HAdV, 9% (95% CI: 5-14) for HRSV, and 9% (95% CI: 6-12) for HPIV. Studies performed in Kenya reported prevalence of 14% (95% CI: 7-25), 10% (95% CI: 6-15), and 9% (95% CI: 7-11) for HAdV, HRSV, and HPIV respectively.	Page 14
**DISCUSSION**			
Summary of evidence	24	A total of 12 studies were eligible for this meta-analysis. Of these, 10 reported HRSV, 8 HPIV, and 9 HAdV. The overall pooled prevalence of HRSV was 11%, 9% for HPIV, and 13% for HAdV. In general, these respiratory viruses were reported in people with ILI, SARI, and/or ARTIs. Overall, 8 (80%) of the reported studies were done in Kenya.	Page15-16
Limitations	25	This systematic review is subject to several limitations. The systematic searches performed in this review were limited to the most accessible and widely used databases of medical literature, including Medline and Global Index Medicus. In addition, the search was complemented with unpublished literature from major public health institutions and research programs in the EAC. We identified several significant sources of heterogeneity in the estimates of pooled prevalence of HRSV, HPIV, and HAdV, including disease severity, age group, and location. Heterogeneity may also be influenced by other factors, including measured factors such as the length of the study period, study design, and laboratory technique. For example, while most studies used highly sensitive and specific diagnostic PCR tests to detect these virusest, studies which used less sensitive diagnostic methods likely underestimated prevalence. Heterogeneity may also have been influenced by unmeasured factors. Selection of participants may also have been different among the studies, likely resulting in higher prevalence among studies in which patients with more severe illness were enrolled, such as SARI as compared to ILI patients. Additionally, the sample size of studies eligible for inclusion in this analysis was small, limiting the power to detect differences, particularly in subgroup analysis. For example, not all countries of the EAC were represented, and Tanzania and Uganda only had one study each. The small sample size may be due to various factors including limited funding, government policy and priorities, the challenges of information sharing, lack of documentation, inaccessibility of databases, and difficulties with publication of data. Further, the presence of extreme values of prevalence introduced computational complexity which limited our ability to report confidence intervals for the I2 values.<br><br>Finally, this review only included studies in which patients were selected based on defined medical conditions such as ILI and SARI or ARTIs in general. Studies that included asymptomatic participants were excluded. Asymptomatic patients would likely have had lower prevalences of these viruses than the symptomatic populations used in our study. Therefore the results of this review cannot be generalized to the general population. The exclusion of asymptomatic cases was considered necessary to avoid overdiagnosis of patients who were colonized or carriers of viruses which may not have ever caused disease.	Page17-18
Conclusions	26	Respiratory illness surveillance programs in the EAC have enhanced the detection of both influenza and non-influenza viruses for over a decade. However, there are no platforms for systematic information sharing in the region. It is vital to establish national and regional information-sharing platforms for non-influenza respiratory	Page 19

		viruses to guide future research, policy, and development. Our findings indicate that human adenoviruses are the most common sources of ILI and SARI other than influenza infection, followed by the human respiratory syncytial virus and parainfluenza virus. Future studies or research could identify the prevalence of HRSV, HPIV, and HAdV using standardized methods and populations to increase comparability among studies and to account for sources of misclassification and heterogeneity. Additional studies should be considered among older populations, among populations from EAC countries from which no data were found, and asymptomatic populations. In addition, the literature search could include additional databases used in biomedical research. Finally, other emerging respiratory pathogens could be studied and further molecular characterization could be carried out to assess transmission.	
**FUNDING**			
Funding	27	NA	Page 20

From: Moher D, Liberati A, Tetzlaff J, Altman DG, The PRISMA Group (2009). Preferred Reporting Items for Systematic Reviews and Meta-Analyses: The PRISMA Statement. PLoS Med 6(7): e1000097. doi:10.1371/journal.pmed1000097

For more information, visit: **www.prisma-statement.org**.
