# Supplementary figures and images for: Prevalence of human respiratory syncytial virus, parainfluenza and adenoviruses in East Africa Community partner states of Kenya, Tanzania, and Uganda: A systematic review and meta-analysis (2007–2020)
Source: PLoS One. 2021 Apr 27;16(4):e0249992. doi: 10.1371/journal.pone.0249992 (PMC8078816; doi:10.1371/journal.pone.0249992)

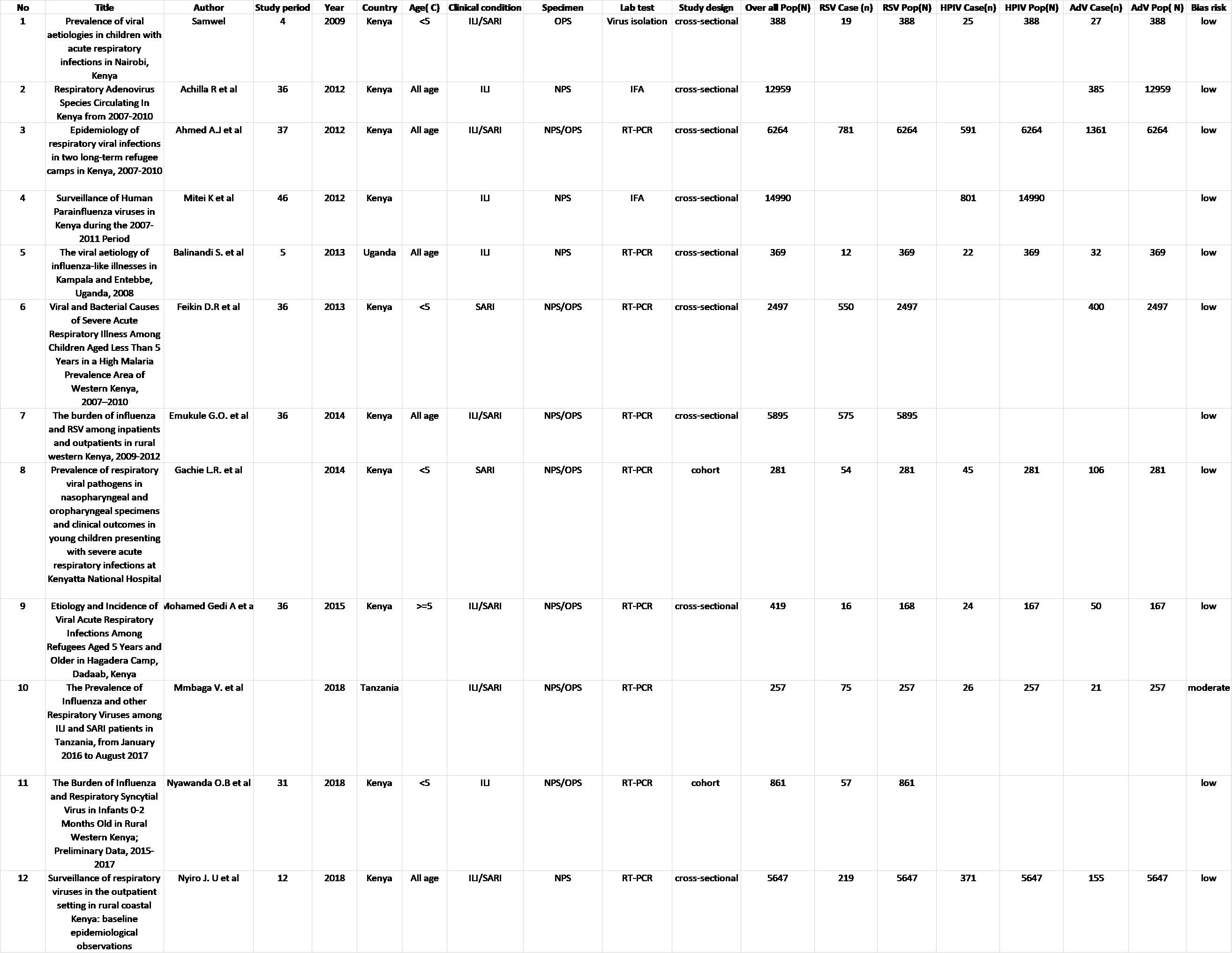

Supplement: S3 File — (TIF) [file pone.0249992.s003.tif]
