# Supplementary material for: Prevalence of human respiratory syncytial virus, parainfluenza and adenoviruses in East Africa Community partner states of Kenya, Tanzania, and Uganda: A systematic review and meta-analysis (2007–2020)
Source: PLoS One. 2021 Apr 27;16(4):e0249992. doi: 10.1371/journal.pone.0249992 (PMC8078816; doi:10.1371/journal.pone.0249992)
Supplement: S4 File — (PDF) [file pone.0249992.s004.pdf]

Individual study bias assessment

No	Author	Study period	Year documentation	Location	Age	Clinical condition	Specimen type	Diagnostic test	Study design	Total score	Bias risk
1	Samwel et al (2009)	1	1	1	1	1	1	1	1	8	low
2	Achilla R et al (2012)	1	1	1	1	1	1	1	1	8	low
3	Ahmed A.J et al (2012)	1	1	1	1	1	1	1	1	8	low
4	Mitei K et al (2012)	1	1	1	0	1	1	1	1	7	low
5	Balinandi S. et al (2013)	1	1	1	1	1	1	1	1	8	low
6	Feikin D.R et al (2013)	1	1	1	1	1	1	1	1	8	low
7	Emukule G.O. et al (2014)	1	1	1	1	1	1	1	1	8	low
8	Gachie L.R. et al (2014)	0	1	1	1	1	1	1	1	7	low
9	Mohamed Gedi A et al (2015)	1	1	1	1	1	1	1	1	8	low
10	Mmbaga V. et al (2018)	0	1	0	0	1	1	1	0	4	moderate
11	Nyawanda O.B et al (2018)	1	1	1	1	1	1	1	1	8	low
12	Nyiro J.U et al (2018)	1	1	1	1	1	1	1	1	8	low

Score category risk of bias

0-2: High 3-5: moderate 6-8 low

Documented and clarity of study characteristic score 1

Absence and or low clarity of study characteristic score 0
